# A Conformational Change in C-Reactive Protein Enhances Leukocyte Recruitment and Reactive Oxygen Species Generation in Ischemia/Reperfusion Injury

**DOI:** 10.3389/fimmu.2018.00675

**Published:** 2018-04-16

**Authors:** Jan R. Thiele, Johannes Zeller, Jurij Kiefer, David Braig, Sheena Kreuzaler, Yvonne Lenz, Lawrence A. Potempa, Florian Grahammer, Tobias B. Huber, M. Huber-Lang, Holger Bannasch, G. Björn Stark, Karlheinz Peter, Steffen U. Eisenhardt

**Affiliations:** ^1^Department of Plastic and Hand Surgery, Medical Center – University of Freiburg, Faculty of Medicine, University of Freiburg, Freiburg, Germany; ^2^College of Pharmacy, Roosevelt University, Schaumburg, IL, United States; ^3^Department of Medicine, University Medical Center Hamburg-Eppendorf, Hamburg, Germany; ^4^Department of Medicine IV, Medical Center – University of Freiburg, Faculty of Medicine, University of Freiburg, Germany; ^5^BIOSS Center for Biological Signalling Studies and Center for Systems Biology (ZBSA), Albert-Ludwigs-University, Freiburg, Germany; ^6^Institute of Clinical and Experimental Trauma-Immunology, University of Ulm, Ulm, Germany; ^7^Baker Heart and Diabetes Institute, Melbourne, VIC, Australia

**Keywords:** C-reactive protein, ischemia/reperfusion injury, reactive oxygen species, therapeutic targets, conformational change, translational medical research, leukocyte recruitment, rat models

## Abstract

**Introduction:**

C-reactive protein circulates as a pentameric protein (pCRP). pCRP is a well-established diagnostic marker as plasma levels rise in response to tissue injury and inflammation. We recently described pro-inflammatory properties of CRP, which are mediated by conformational changes from pCRP to bioactive isoforms expressing pro-inflammatory neo-epitopes [pCRP* and monomeric C-reactive protein (mCRP)]. Here, we investigate the role of CRP isoforms in renal ischemia/reperfusion injury (IRI).

**Methods:**

Rat kidneys in animals with and without intraperitoneally injected pCRP were subjected to IRI by the time of pCRP exposure and were subsequently analyzed for monocyte infiltration, caspase-3 expression, and tubular damage. Blood urea nitrogen (BUN) was analyzed pre-ischemia and post-reperfusion. CRP effects on leukocyte recruitment were investigated *via* intravital imaging of rat-striated muscle IRI. Localized conformational CRP changes were analyzed by immunohistochemistry using conformation specific antibodies. 1,6-bis(phosphocholine)-hexane (1,6-bisPC), which stabilizes CRP in its native pentameric form was used to validate CRP effects. Leukocyte activation was assessed by quantification of reactive oxygen species (ROS) induction by CRP isoforms *ex vivo* and *in vitro* through electron spin resonance spectroscopy. Signaling pathways were analyzed by disrupting lipid rafts with nystatin and subsequent ROS detection. In order to confirm the translational relevance of our findings, biopsies of microsurgical human free tissue transfers before and after IRI were examined by immunofluorescence for CRP deposition and co-localization of CD68^+^ leukocytes.

**Results:**

The application of pCRP aggravates tissue damage in renal IRI. 1,6-bisPC reverses these effects *via* inhibition of the conformational change that leads to exposure of pro-inflammatory epitopes in CRP (pCRP* and mCRP). Structurally altered CRP induces leukocyte–endothelial interaction and induces ROS formation in leukocytes, the latter can be abrogated by blocking lipid raft-dependent signaling pathways with Nystatin. Stabilizing pCRP in its native pentameric state abrogates these pro-inflammatory effects. Importantly, these findings are confirmed in human IRI challenged muscle tissue.

**Conclusion:**

These results suggest that CRP is a potent modulator of IRI. Stabilizing the native pCRP conformation represents a promising anti-inflammatory therapeutic strategy by attenuation of leukocyte recruitment and ROS formation, the primary pathomechanisms of IRI.

## Introduction

Ischemia/reperfusion injury (IRI) is an inflammatory response that occurs when tissue is reperfused following a prolonged period of ischemia (1). The main responsible pathomechanisms of this inflammation are often overshooting leukocyte activation (2, 3), complement activation (4), and generation of reactive oxygen species (ROS) (5–7) that lead to the release of pro-inflammatory cytokines and increased vascular permeability and consequently result in tissue damage. Renal IRI is inevitable in many clinical situations such as renal transplantation, vascular surgery (8), acute ischemic renal injury, and delayed graft function (9). Due to the relatively limited understanding of the pathophysiology, there is to date no specific treatment of this devastating clinical condition. The current research, therefore, addresses the major medical need to identify new therapeutic approaches to IRI.

Recently, we have been able to show that C-reactive protein (CRP), an acute phase reactant that is elevated after tissue injury, undergoes conformational changes from its circulating native pentameric isoform (pCRP) to a bioactive conformation (pCRP*). pCRP* binds complement C1q and activates the classical complement pathway. It then further dissociates into monomeric CRP subunits [monomeric C-reactive protein (mCRP)], which exert further pro-inflammatory actions before they are cleared by phagocytes (10–14). These conformational changes are mediated by bioactive lipids (15) on activated or damaged cells or platelets (10, 16). Conformation-specific antibodies can detect a neo-epitope (that is, residues 199–206 of CRP become accessible), which is present on both pCRP* and mCRP, but not on pCRP (10, 17). This neo-epitope mediates most of the pro-inflammatory CRP effects (12, 18). pCRP* is the major pro-inflammatory isoform *in vivo*, but exists only on biological surfaces and thus cannot be purified for *in vitro* use. Therefore, mCRP is commonly used as surrogate to study pro-inflammatory pCRP* effects *in vitro* as it presents the same bioactive epitopes. mCRP leads to increased monocyte activation, adhesion, and transmigration, as well as formation of ROS (10) and activation of the complement system (12), which represent major pathophysiological factors contributing to tissue injury in IRI. Thus, we hypothesized that the conformational change of pCRP and the consecutive aggravation of inflammation might be a pathophysiological mechanism by which inflammation is regulated and localized in IRI and thus represents a therapeutic target to reduce tissue damage in IRI.

## Materials and Methods

### Reagents

Human pCRP was purchased from Calbiochem (Nottingham, UK; purified from human ascites) and was dialyzed against Dulbecco’s phosphate buffered saline with Ca^2+^/Mg^2+^ (D-PBS) (ThermoFisher Scientific) to prevent potential contaminations and tested as described before (11, 12). 1,6-bisPC was synthesized by Syngene International, Bangalore, India. Lipopolysaccharide (LPS) from *E. coli* serotype O127:B8 for intravital microscopy was obtained from Sigma-Aldrich. As described previously, we utilized and prepared mCRP (1 mg/ml) in soluble, citraconylated form (19). Conformation-specific CRP antibodies clone 8D8 and 9C9 were kindly provided by Dr. Larry Potempa (College of Pharmacy, Roosevelt University, Schaumburg, IL, USA) (20).

### Animals

Male Wistar rats were purchased from Charles River Research Models and Services (Sulzfeld, Germany). For the renal IRI-model, all rats were 6 weeks old and body weight was between 180 and 220 g. Male Wistar rats for intravital microscopy were selected and handled as previously described (11). Animals were housed in light controlled rooms (12 h light/dark cycle) and allowed access to food and water *ad libitum*. This study was carried out in accordance with the recommendations of the animal ethic committee of the University of Freiburg Medical Center, Germany. The protocol was approved by the animal ethic committee of the University of Freiburg Medical Center, Germany.

### Human Studies

For immunohistology of human ischemia/reperfusion-injured tissue, biopsies of 15 patients receiving free muscle flap reconstruction of posttraumatic soft tissue defects of the lower extremity were taken between September 2008 and March 2010. Informed consent was obtained from each patient. The study was approved by the ethic committee of the University of Freiburg Medical Center (Application number: 67/08) and conducted in accordance with the declaration of Helsinki.

### Renal Ischemia/Reperfusion-Injury Model

Prior to surgery, 30 Wistar rats were randomly allocated to one of five designated groups; (1) sham-operated controls receiving flank incisions without renal clamping. Animals received i.p. vehicle D-PBS solution treatment; (2) IRI-treated rats were subjected to the surgical procedure described hereafter. IRI rats received i.p. 500 µl D-PBS application; (3) IRI + pCRP-treated rats: the same surgical procedure as in group (2) was performed. Animals received i.p. pCRP application in a 25 µg/ml serum concentration instead of D-PBS; (4) IRI + pCRP + 1,6-bisPC-treated group: as in group (3) rats received i.p. pCRP application in a 25 µg/ml serum concentration. pCRP was incubated with 1,6-bisPC (1:100 molar ratio) before administration; (5) IRI + 1,6-bisPC-treated group: the same surgical procedure as in group (2) was performed. Animals received i.p. 1,6-bisPC application only (*n* = 6 per group).

### Experimental Protocol of Renal IRI

The surgical procedure was a modification of the renal IRI-model described by Delbridge et al. (21). A bilateral ischemic AKI modification was considered more relevant to human pathological conditions (22). In brief, Wistar rats were anesthetized with 1.5–2 vol% isoflurane (Abbott, Wiesbaden, Germany) *via* silicone mask and received subcutaneous buprenorphine (0.05 mg/kg body weight) (23) for pain relief. Buprenorphine is a convenient option for analgesics in IRI-models since it is long-acting with a high therapeutic index and metabolized in the liver (24). Adequate depth of anesthesia to commence following surgery was achieved by loss of reflexes to toe pinch test and distinct slowing of respiratory rate. An eye lubricating ointment (Bepanthen, Bayer Vital GmbH, Leverkusen, Germany) was used to avoid postoperative blinding of the rat. Animals were placed in lateral recumbency on a heated surgical table to maintain core body temperature at 37°C (anal probe-controlled) to avoid effects of the body temperature on the severity of IRI (21, 22). Both renal pedicles were exposed *via* two paravertebral flank incisions and clamped with nontraumatic micro vessel clips for 45 min followed by 24 h reperfusion. A gradual change in color from light red to dark purple served as a surrogate parameter for a successfully induced ischemia of the kidney. The kidneys were embedded in saline solution soaked gazes during the period of exposure. Simultaneously, weight-adapted volume of group-corresponding solution was administered intraperitoneally. Serum volume was estimated as described before (11) as a function of the body weight (25). A second bolus was injected i.p. after 12 h of reperfusion and constant serum levels of pCRP were verified by immunologic turbidity measurements. Immediately after surgery, subcutaneous saline supplementation was given to avoid dehydration of the rats. All microsurgical procedures were conducted using a stereo microscope (Stemi 2000, Carl Zeiss).

### Measurement of Blood Urea Nitrogen (BUN)

Renal function was assessed by BUN concentration (26, 27). Blood samples were obtained *via* lateral tail vein sampling in micro tubes with clotting activator (Micro tube 1.3 ml Z, Clotting Activator/Serum, Sarstedt, Nümbrecht, Germany). Preparation of clotted blood samples was conducted using a precooled tabletop centrifuge (Eppendorf centrifuge 5427R, Eppendorf AG, Hamburg, Germany). To receive designated serum, probes were centrifugated at 2,000 × *g* for 10 min at 5°C. Measurement of BUN concentration was performed using cobas 8000 modular analyzer (cobas 8000 modular analyzer series, Roche, Basel) by the central laboratories of the University Medical Center, Freiburg. To avoid invalidation, samples that showed macroscopic levels of hemolysis were excluded.

### Immunostaining and Histomorphological Evaluation

Immunohistochemistry and histomorphological evaluation of the renal tissue was performed on formalin-fixed paraffin-embedded renal tissue sections (5 µm thick serial sections). Previously, both kidneys were flushed till bloodlessness with D-PBS followed by 4% formalin for fixation. Kidneys were then excised and examined in blinded fashion by two researchers using a Zeiss microscope (Carl Zeiss Microscopy Axio Imager.M2, Germany). Staining was performed as described previously (17, 28, 29) with minor modifications. Paraffin-embedded sections were de-paraffinized in xylol, rehydrated, and boiled for 20 min in concentrated citric acid (pH 6.0). Antigen unmasking for anti-monocyte detection was done by application of pepsin solution (Digest-All™ 3, life technologies) at room temperature for 20 min (30). Histomorphological changes were evaluated on Periodic acid–Schiff stained sections by quantitative measurement of tubulointerstitial injury, which was assessed by loss of tubular brush border and cast formation following an established protocol (31, 32). In brief, the morphological assessment was scaled in five steps: with not present (0), mild (1), moderate (2), severe (3) to very severe (4). Transmigrated leukocytes were detected by anti-monocyte/macrophage antibody clone ED-1 (Millipore, Billerica, MA, USA) in a 1:100 dilution and renal inflammation was evaluated by counting ED-1^+^ cells in 20 randomized areas of interest of the renal cortex at ×200 magnification. The number of apoptotic cells was evaluated using anti-caspase-3 antibody (Novus Biologicals, Abingdon, UK) in a 1:1,000 dilution (33). Sections were counterstained with Mayer’s hematoxylin. Negative immunocontrols were issued by sections to which primary antibodies had not been added. Each parameter was determined on at least five different animals per group. As a proof of concept, detection of human CRP on the renal tissue sections was performed using anti-pCRP*/mCRP antibody 9C9 (1:100 dilution). The immunostaining for CRP conformations in cremaster muscle was conducted following the surgical procedure described below. The cremaster muscle was excised, snap-frozen, and conserved in tissue freezing medium (Leica Microsystems, Nussloch, Germany). Tissue samples were cut serially in horizontal direction into 6 µm sections. For conformation specific detection of pCRP, we utilized antibody clone 8D8 and antibody clone 9C9 was used for the detection of conformationally altered CRP (pCRP*/mCRP) (1:100 dilution) (34). Immunostaining proceeded as described in earlier work (10).

### Intravital Microscopy Studies of Rat Cremaster Muscle

As previously described (11, 12) and published in a detailed protocol (35), leukocyte–endothelial interaction was observed in the microcirculation of the cremaster muscle in male Wistar rats (weighing 120–180 g) using intravital microscopy. Briefly, the rats were anesthetized with 1.5–2 vol% isoflurane and volume controlled ventilated *via* tracheotomy (Servo Ventilator 900C, Maquet, Rastatt, Germany; settings: frequency 35–45 breaths/min, tidal volume 4.5–5 ml, FiO_2_ 0.35–0.5). Vital parameters (heart rate, mean arterial pressure, blood gases) were monitored through a cannulated carotid artery. Intravenous injection of rhodamine 6G (0.4 mg/kg body weight, Sigma-Aldrich) (36) *via* an established jugular vein port stained the circulating leukocytes and enabled for intravital tracking. After externalization of the cremaster muscle and visualization of the cremasteric microcirculation, leukocyte rolling and adherence was assessed. Leukocyte rolling was defined as significantly slower moving leukocytes compared to erythrocytes within the same vessel. Adherent leukocytes remained stationary for 20 s or more.

### Western Blot Analysis

Native Western blot analysis was conducted for CRP detection in the cremaster muscle as described previously (11). Briefly, muscle tissue was excised and homogenized on ice using a high-power disperser (Ultra-Turrax^®^ IKA, Staufen, Germany). The purification of the cell lysates was determined by a BCA protein assay kit (Sigma-Aldrich) and portioned. After the separation by SDS gel electrophoresis and the transfer to Hybond ECL nitrocellulose membranes (GE Healthcare, Munich, Germany), samples were probed with anti-pCRP*/mCRP antibody for 1 h at RT. To ensure equilibration, we used monoclonal antibodies against GAPDH (abcam, Cambridge, UK). An anti-mouse horseradish peroxidase-conjugated secondary antibody (Dianova, Hamburg, Germany) was utilized for detection using enhanced chemiluminescence (ECL, GE Healthcare) and conserved on Hyperfilm ECL (GE Healthcare). Cell lysis and Western blotting buffer were used as described earlier (11).

### Electron Spin Resonance (ESR) Spectroscopy for the Study of Leukocyte-Derived ROS

For *ex vivo* experimental procedures, we utilized ESR spectroscopy to identify and quantify ROS formation in rat leukocytes. CMH (1 mM, 1-hydroxy-3-methoxycarbonyl-2,2,5,5-tetramethylpyrrolidine, Noxygen, Elzach, Germany) was adopted as a spin label suitable for biological utilization as described before (37). After the intravital experimental procedure, whole blood samples were collected in EDTA tubes by cannulation of the abdominal aorta and mononuclear cells [peripheral blood mononuclear cells (PBMC) and polymorphonuclear cells (PMN)] were isolated by Ficoll density gradient (Bicoll Separating Solution, Biochrom, Berlin, Germany; density 1.077 kg/m^3^) centrifugation as described before (11). MiniScope MS 200 ESR Spectrometer (Magnettech, Berlin, Germany) was used for measurements with following instrument settings: center field, 3,340G; sweep wide, 60G; sweep time, 5 ms over 10 scans; modulation amplitude, 2.4G; microwave power, 10 mW. Positive controls were conducted by cremasteric superfusion with LPS in concentration 1 µg/ml. For the pCRP/mCRP groups, 25 µg/ml pCRP or mCRP was administrated intravenously. Blood samples were taken 60 min after the treatment.

For *in vitro* experiments, whole blood samples were collected from rats and healthy human donors and PBMC and PMN were isolated as described above. The cell suspensions were then incubated at 37°C for 30 min with PBS only (control), LPS (50 µg/ml), pCRP (10 µg/ml), and mCRP (10 µg/ml), respectively.

### Statistical Analysis

Statistical analysis was performed using GraphPad Prism v7.0 software (GraphPad Software, San Diego, CA, USA). For comparison of two groups, a two-tailed *t*-test was employed. Experimental data were compared using one-way ANOVA to compare effects of different treatments, if more than two groups were compared. In case of significance, Turkey’s test was performed for pairwise comparison. To analyze treatment effects over time, we performed a two-way repeated measures (mixed model) ANOVA with the fixed factors “time,” “treatment,” and the corresponding interaction term. In case of a non-significant interaction and significant treatment effect, pairwise Bonferroni adjusted comparisons were performed at each time point. Significant results for both two-way repeated measures (mixed model) ANOVA and Bonferroni *post hoc* tests are presented. A *p*-value <0.05 statistical significance level was accepted. All data are expressed as scatter plot with mean ± SEM.

## Results

### CRP Increases Tissue Damage in Renal IRI

**Figure 1:**
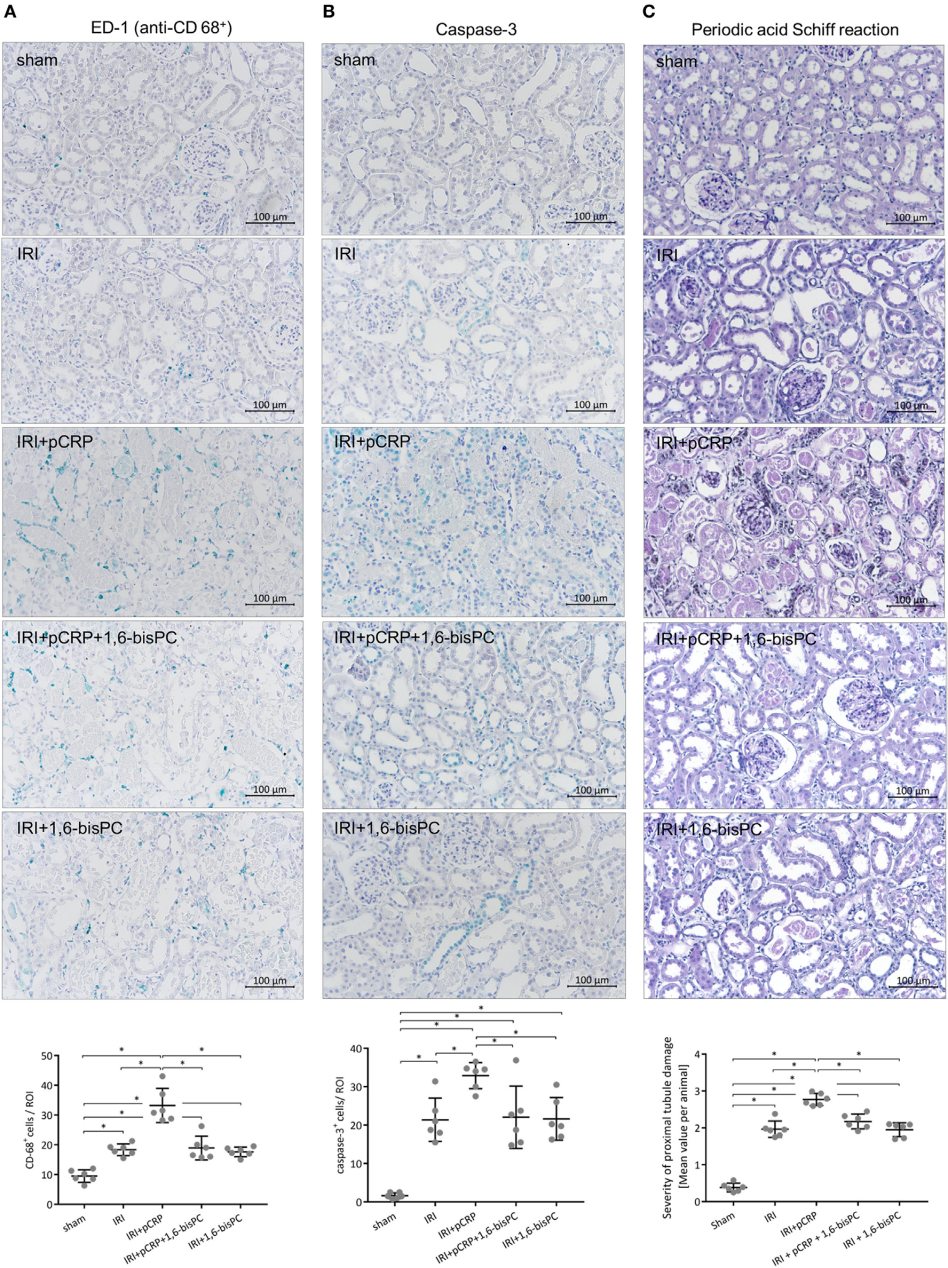

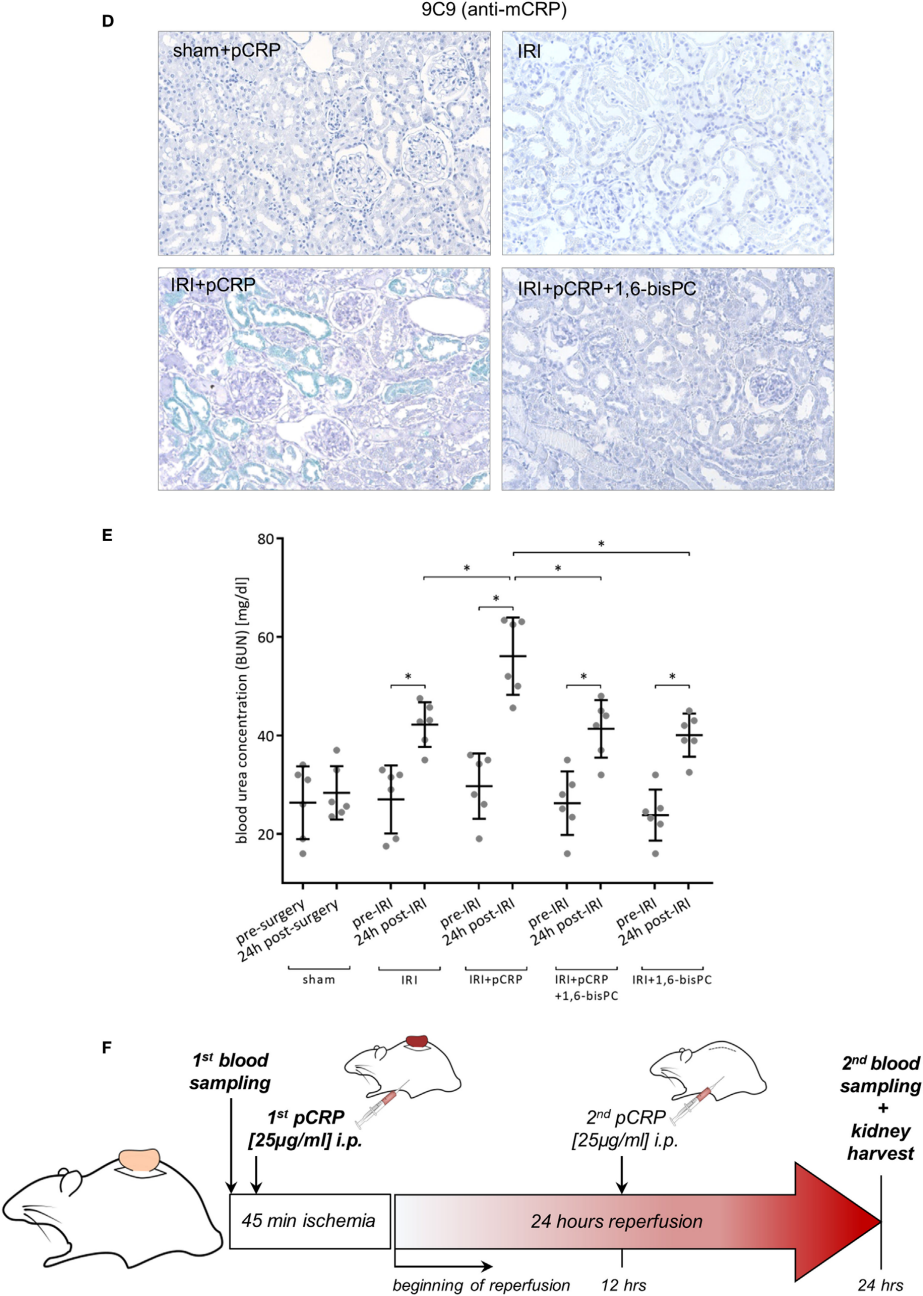
C-reactive protein (CRP) aggravates renal ischemia/reperfusion injury (IRI) in rats. **(A)** Immunohistochemical detection of CD68^+^ cells in the renal cortex and quantification of the results. Antibody binding was detected with the HistoGreen substrate kit. CD68^+^ cells were quantified in randomly chosen areas of the renal cortex at ×200 magnification. Given are mean cell counts/area per animal. *p < 0.05 for IRI + pentameric C-reactive protein (pCRP) vs. IRI, IRI + pCRP + 1,6-bisPC, and IRI + 1,6-bisPC. Values are mean ± SEM; *n* = 6. The number of CD68^+^ cells transmigrating in the I/R-injured tissue is significantly increased under pCRP application; stabilization of pCRP with 1,6-bisPC abrogates this effect. **(B)** Localization and quantification of caspase-3 protein in the tubular epithelium. Anti-caspase-3 antibody and HistoGreen staining of apoptotic cells in the renal tubules. Given are counts of positive cells. Randomly selected areas of the renal cortex were evaluated from each sample. *p < 0.05 for IRI + pCRP vs. IRI/IRI + pCRP + 1,6-bisPC/IRI + 1,6-bisPC. Values are mean ± SEM; *n* = 6. CRP significantly increases the number of apoptotic cells in IRI. This is inhibited by addition of 1,6-bisPC. **(C)** Histomorphological evaluation of renal tissue after IRI using Periodic acid–Schiff (PAS) staining. A minimum of ten fields per slide was examined at ×200 magnification. Shown are mean values per animal for the severity of proximal tubule damage. *p < 0.05 for IRI + pentameric C-reactive protein (pCRP) vs. IRI/IRI + pCRP + 1,6-bisPC/IRI + 1,6-bisPC. Not significant for IRI vs. IRI + pCRP + 1,6-bisPC and IRI vs. IRI + 1,6-bisPC. **(D)** Immunohistochemical detection of CRP neo-epitopes with anti-pCRP*/monomeric C-reactive protein (mCRP) antibody 9C9 and HistoGreen after IRI and i.p. application of pCRP ± 1,6-bisPC compared with IRI without pCRP application and sham-operated kidneys with pCRP application. After i.p. application of pCRP, conformationally altered CRP is deposited in renal tubules. This is not seen when pCRP is pre-stabilized with 1,6-bisPC. **(E)** Analysis of blood urea nitrogen. Blood samples were taken before ischemia/surgery and after the 24 h reperfusion period or 24 h after surgery, respectively. Renal IRI induces a significant increase in BUN, a functional hallmark of renal function in acute kidney injury. CRP-dissociation aggravates the functional tissue damage, 1,6-bisPC prevents pro-inflammatory alterations of CRP and partially preserves kidney function. *p < 0.05 for IRI + pCRP vs. IRI/IRI + pCRP + 1,6-bisPC/IRI + 1,6-bisPC; *p < 0.05 for all groups pre-ischemia vs. postischemia. **(F)** Flow chart representing the experimental protocol of the renal IRI model.

Rat renal IRI was quantified by determination of BUN, immunohistological staining for caspase-3 and monocytes as well as evaluation of morphological changes after reperfusion. All experimental readouts consistently demonstrate that intraperitoneal (i.p.) injection of pCRP potentiates renal IRI leading to elevated BUN, increased caspase-3 expression, and aggravated IRI-specific alterations in tissue morphology. A significant increase in the number of infiltrated monocytes could be detected (Figures 1A–E). Immunohistological staining with anti-pCRP*/mCRP-antibody 9C9 demonstrates that the injected pCRP is not deposited in healthy renal tissue, however, accumulates in IRI-damaged tubules in its conformationally altered isoforms (Figure 1D).

### CRP Aggravates Leukocyte Recruitment in IRI, but Does Not Show Intrinsic Pro-Inflammatory Potential in Sham-Operated Animals

In a model of IRI, leukocyte–endothelial interaction in the microcirculation of the rat cremaster muscle was observed *via* intravital microscopy.

**Figure 2:**
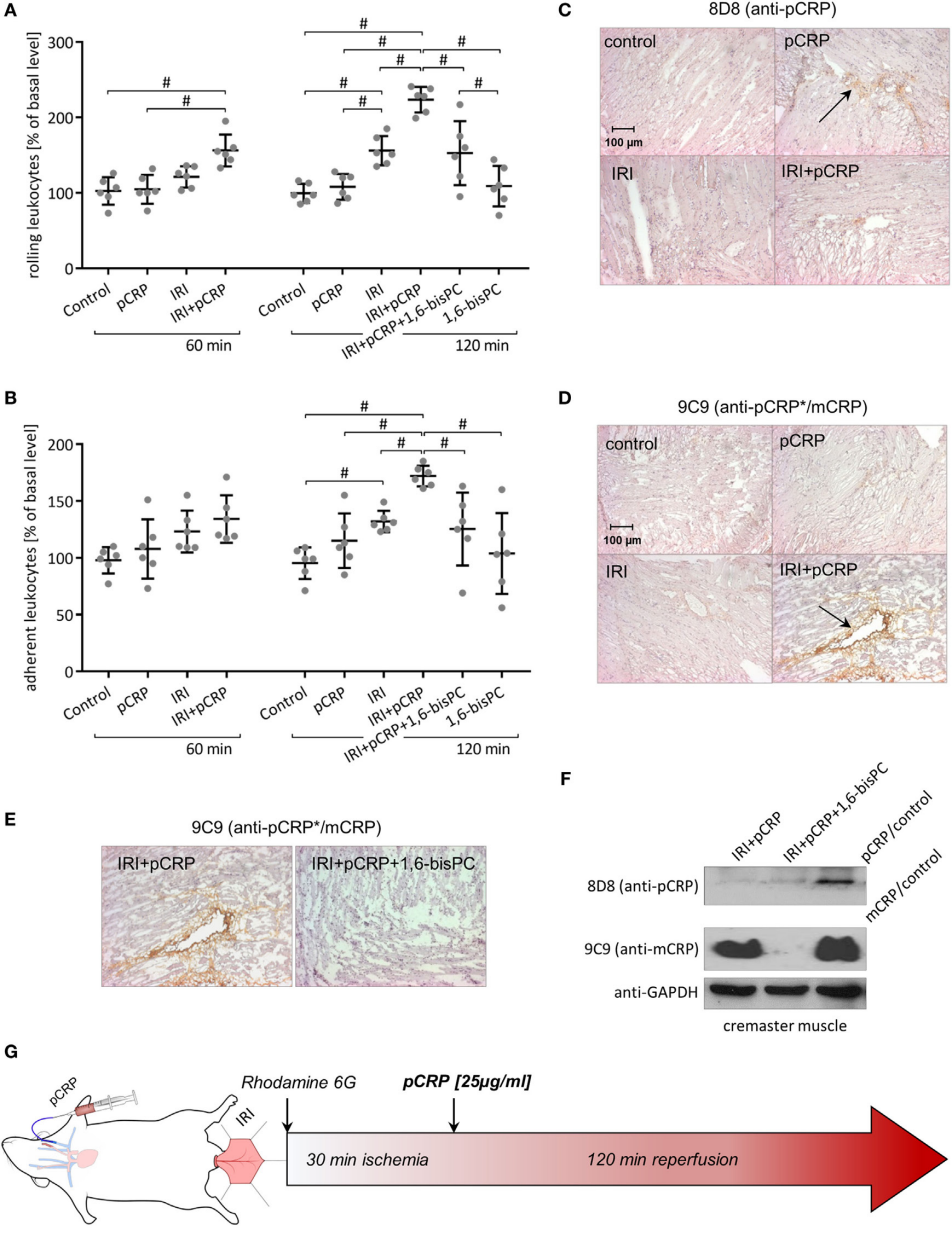
C-reactive protein (CRP)-induced aggravation of ischemia/reperfusion injury (IRI) is controlled by a localized conformational change regulating leukocyte recruitment. **(A,B)** Intravital microscopy of rat cremasteric postcapillary venules to determine leukocyte–endothelial interaction in IRI ± i.v. injection of pentameric C-reactive protein (pCRP) (25 µg/ml). Leukocytes were labeled with Rhodamine 6G. Counts at 0 min was set to 100%. Values are mean ± SEM of 10–14 observed venules in 6 rats. ^#^*p* < 0.05. i.v. application of pCRP aggravates IRI and significantly increases leukocyte rolling **(A)** and adhesion **(B)**. 1,6-bisPC masks the pro-inflammatory potential of CRP in IRI and induces a significant decrease in leukocyte rolling **(A)** and adhesion **(B)** compared to the IRI + pCRP group. 1,6-bisPC alone has neither pro- nor anti-inflammatory potential. **(C,D)** Immunohistochemical staining of the cremaster muscle with DAB after IRI ± i.v. application of pCRP. Clone 8D8 **(C)** was used to detect pCRP and clone 9C9 **(D)** was used to detect conformationally altered CRP. Representative results are shown (*n* = 6 for each sample). After i.v. application, pCRP can be detected in the cremasteric tissue (arrow). Staining gets less pronounced in IRI. Neo-epitope expressing CRP is strongly detectable after pCRP injection in IRI (arrow), though trace amounts are detected in healthy tissue. IRI induces an alteration of the CRP conformation and a deposition in the inflamed tissue. **(E)** Immunohistochemical staining of the cremaster muscle with DAB after IRI and i.v. application of pCRP ± 1,6-bisPC. Stabilization of pCRP by 1,6-bisPC abrogates the formation and deposition of conformationally altered CRP. **(F)** Western blot analysis of native PAGE (1/20 SDS) of I/R-injured rat cremaster muscle tissue stained for mCRP with antibody clone 9C9 and 8D8. In the I/R-injured muscle tissue, altered CRP accumulates after the dissociation of pCRP. 1,6-bisPC stabilizes pCRP in its native conformation and, therefore, altered CRP is not detectable in 1,6-bisPC-treated rats. **(G)** Flow chart of the intravital experimental protocol.

Moderate inflammatory response was induced by ischemic occlusion of the cremaster muscle for 30 min, resulting in an increase in leukocyte rolling that reaches statistical significance after 60 min and in leukocyte adhesion by 120 min. pCRP infusion has no impact on leukocyte recruitment in the resting muscle tissue. However, tissue alteration through IRI results in a marked aggravation of the IRI-induced inflammatory response through application of pCRP. Thus, the number of rolling leukocytes in IRI significantly increases after 60 min and more than doubles to the end of the imaging period (Figure 2A). Likewise, leukocyte adhesion increases in IRI in the presence of pCRP; however, effects are timely delayed compared to rolling leukocytes. Values do not reach statistical significance compared to the control until 120 min after infusion of pCRP (Figures 2B,G).

### In IRI of Striated Muscle Tissue, Conformationally Altered CRP Is Deposited in the Interstitial Space

Rat cremaster muscle samples were examined for deposition of CRP by immunohistochemistry after intravital imaging using conformation specific antibodies. Staining reveals that the infused native pCRP can only weakly be detected in the interstitial space of healthy tissue by anti-pCRP 8D8 antibodies (Figure 2C). This is in contrast to the IRI-mediated inflamed muscle tissue, where total amounts of CRP deposition increases (Figure 2D). Here, CRP is in large parts detectable by anti-pCRP*/mCRP 9C9 antibodies and thus deposited in its conformationally altered isoforms.

### Blocking the CRP Conformational Change Abrogates Pro-Inflammatory Effects

The small molecule inhibitor 1,6-bisPC is able to stabilize pCRP in a decameric confirmation, thereby inhibiting the pro-inflammatory conformational change (pCRP*/mCRP) (11). Here, we show that the decameric stabilization prevents deposition of CRP in IRI-altered tubules of the kidney (Figure 1D). Conformation-specific staining for CRP as well as native Western blotting of the cremasteric tissue (Figures 2C–F) reveals that 1,6-bisPC prevents the dissociation of pCRP in IRI, thereby impending the deposition of pCRP*/mCRP in the interstitial space. This in turn masks the pro-inflammatory contribution of CRP to the inflammatory reaction in renal IRI as well as in IRI of striated muscle tissue. Significant decreases in caspase-3 expression, monocyte infiltration, and tubular damage clearly demonstrate the protective effect of 1,6-bisPC in the CRP-induced tissue damage in renal IRI (Figures 1A–C). This is also reflected in the significant decrease in BUN under 1,6-bisPC compared to the elevated concentrations in IRI under CRP alone (Figure 1E). 1,6-bisPC does not only soften the CRP driven inflammatory response in IRI detected after a 24 h reperfusion period but also modulates the immediate inflammatory reaction after ischemia. This is reflected in a mitigated leukocyte–endothelial interaction in the microcirculation of the IRI-challenged cremasteric tissue after pretreatment of pCRP with 1,6-bisPC, showing significant decreases in leukocyte rolling and adhesion after 120 min. 1,6-bisPC alone, however, shows neither pro- nor anti-inflammatory potential (Figures 2A,B).

### Exposition of Pro-Inflammatory Neo-Epitopes in Inflamed Tissue Can Be Mimicked by Pre-Dissociated CRP

**Figure 3:**
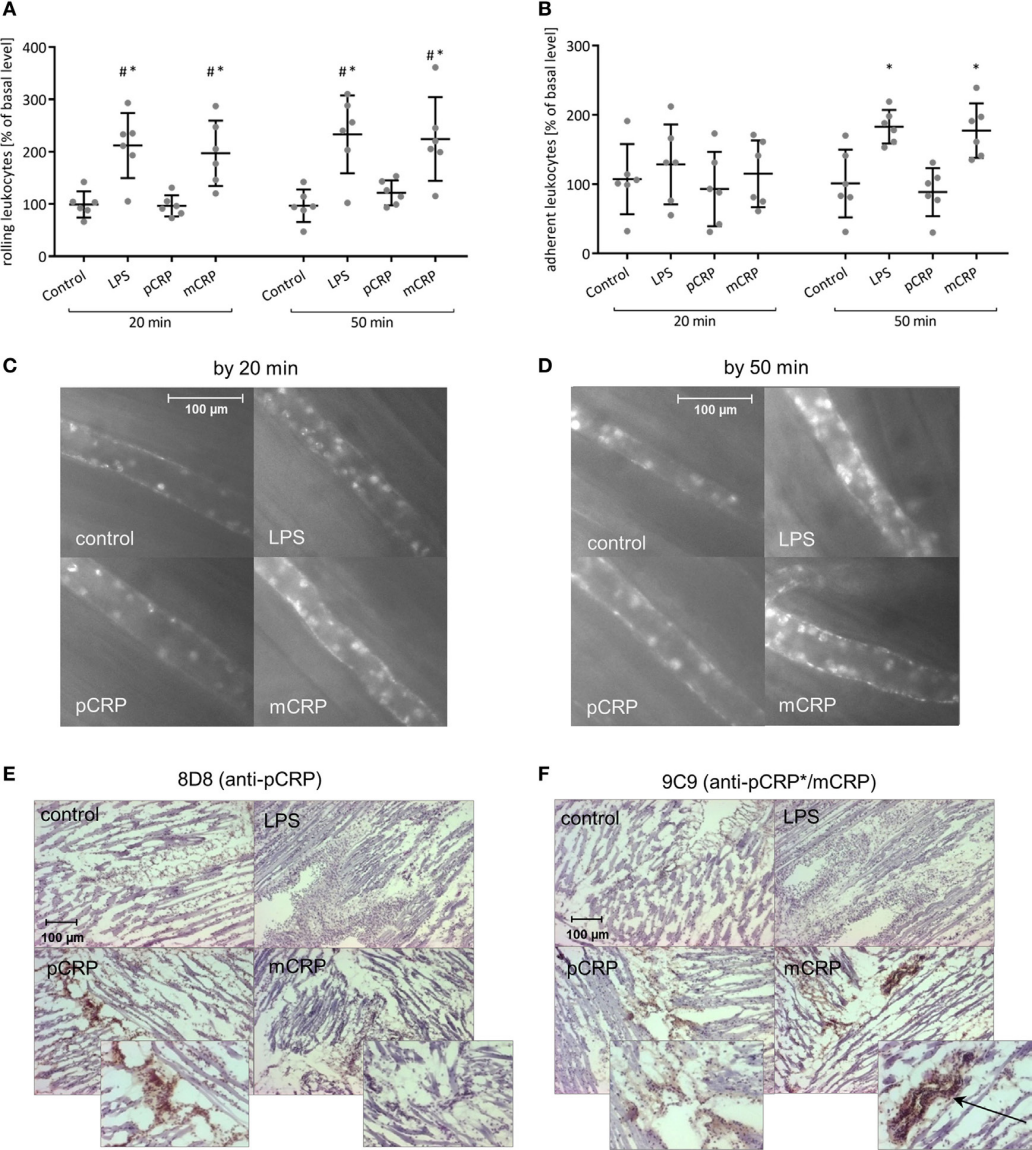
Monomeric C-reactive protein (mCRP) is the pro-inflammatory tissue-appealing isoform of C-reactive protein (CRP). **(A,B)** Leukocyte–endothelial interaction under i.v. injection of either mCRP or pentameric C-reactive protein (pCRP) (25 µg/ml) quantified by intravital microscopy. Superfusion of the cremaster muscle with lipopolysaccharide (LPS) (1 µg/ml) served as positive control. Counts at 0 min was set to 100%. Values are mean ± SEM of 10–14 observed postcapillary venules in six rats. ^#^*p* < 0.05 compared to the control; *p < 0.05 compared to the pCRP group. mCRP significantly increases leukocyte rolling **(A)** and leads to a rapid increase of leukocyte adhesion **(B)** during the course of the experimental protocol whereas pCRP shows no significant effect. Images shown under **(C,D)** are typical venules of the four groups after 20 and 50 min. **(E,F)** Immunohistochemical staining of the cremaster muscle with DAB after i.v. application of pCRP (25 µg/ml) and mCRP (25 µg/ml). Clone 8D8 was used to detect pCRP **(E)** and clone 9C9 was used to detect neo-epitope expressing CRP (pCRP*/mCRP) **(F)**. Representative results are shown (*n* = 6 for each sample). Trace amounts of pCRP can be detected in the cremasteric tissue in the pCRP group. mCRP application results in a pronounced positive staining for mCRP particularly around cremasteric vessels (arrow).

In order to analyze the pro-inflammatory potential of CRP when neo-epitopes are exposed, we used pre-dissociated mCRP in rat intravital microscopy. Superfusion of the cremaster muscle with (LPS) (1 µg/ml) served as positive control. mCRP but not pCRP infusion leads to a rapid increase of leukocyte rolling in the cremasteric microcirculation with significant differences after 20 min compared to the control and to the pCRP group (Figures 3A,C). This is in line with the increase in the number of adherent leukocytes under mCRP, though values do not reach statistical significance before 50 min (Figures 3B,D). Subsequent conformation-specific immunohistochemical staining of the cremaster muscle reveals that the infused mCRP is extensively deposited in and around cremasteric vessels. pCRP can only be detected in trace amounts in the cremasteric tissue after pCRP infusion (Figures 3E,F).

### mCRP Induces Respiratory Burst in Rat and Human Leukocytes Which Is Mediated by Lipid Rafts in PBMC

**Figure 4:**
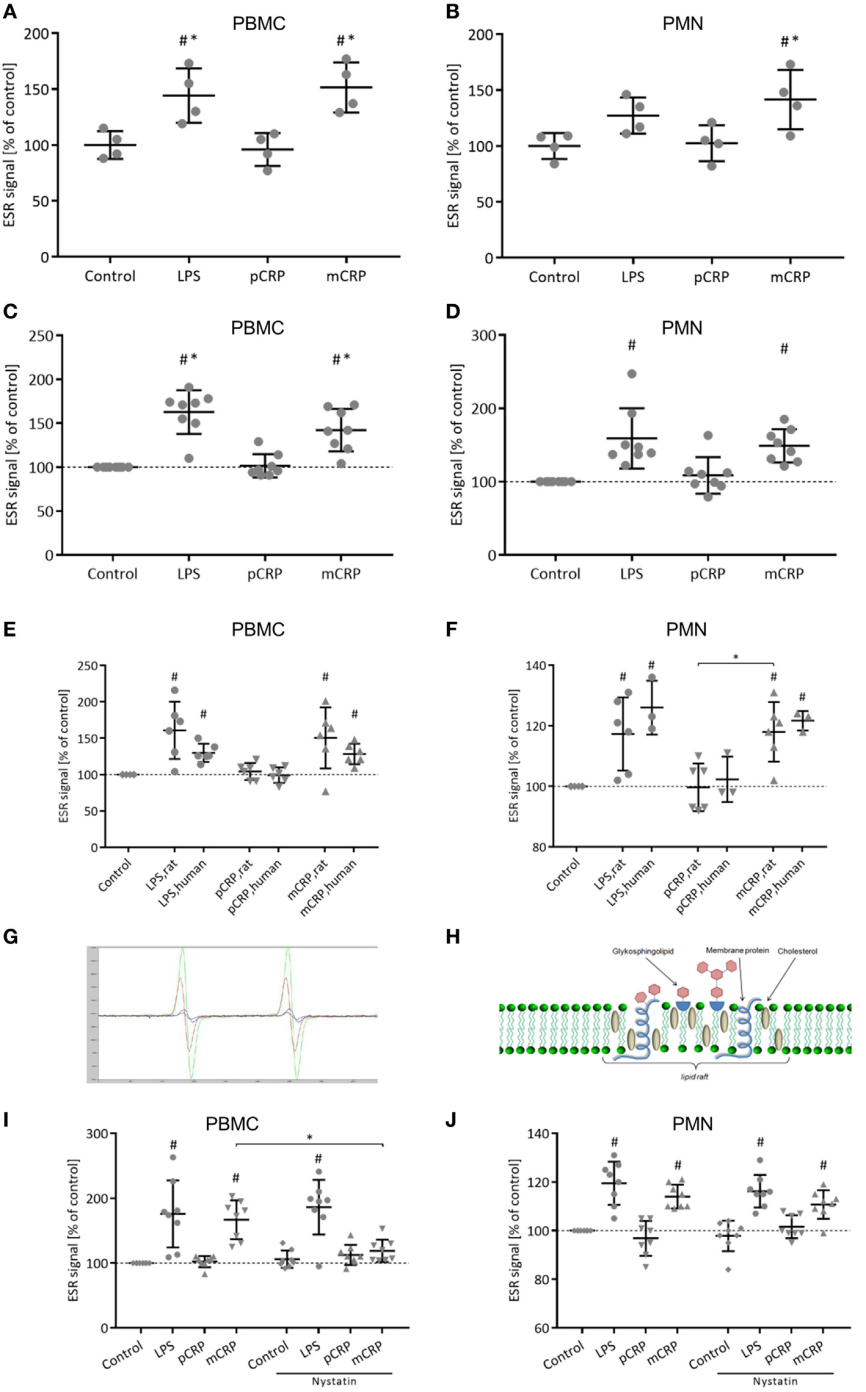
Monomeric C-reactive protein (mCRP) increases reactive oxygen species (ROS) formation in rat leukocytes. This is reproducible for human leukocytes. Nystatin disrupts the pro-inflammatory signaling of mCRP in rat peripheral blood mononuclear cells (PBMC). **(A,B)** *Ex vivo* electron spin resonance (ESR) detection using CMH (25 µg/ml) as spin label in rat PBMC and polymorphonuclear cells (PMN) after *in vivo* treatment with p-/mCRP (25 µg/ml). Values are mean ± SEM of four different rats. ^#^*p* < 0.05 compared to the control group. *p < 0.05 compared to the pentameric C-reactive protein (pCRP) group. mCRP induces a significant stimulation of ROS in PBMC **(A)** as well as in PMN **(B)**. Cremasteric superfusion with lipopolysaccharide (LPS) (1 µg/ml) served as control. **(C,D)** *In vitro* ESR detection in isolated rat PBMC and PMN after incubation with p-/mCRP (10 µg/ml). LPS (50 µg/ml) served as positive control. Values are mean ± SEM of eight different rats. ^#^*p* < 0.05 compared to the control group. *p < 0.05 compared to the pCRP group. mCRP induces a significant stimulation of ROS in PBMC as well as in PMN. **(E,F)** *In vitro* ESR detection in isolated human and rat PBMC/PMN after incubation with p-/mCRP (10 µg/ml). LPS (50 µg/ml) served as positive control. Values are mean ± SEM of at least three different rats/human donors. ^#^*p* < 0.05 compared to the control group. *p < 0.05 for pCRP vs. mCRP. ROS stimulation by mCRP in rat leukocytes can be reproduced in human leukocytes. **(G)** Representative spectra of electron spin resonance (ESR) spectroscopy. **(H)** Schematic drawing of a lipid raft. **(I,J)** *In vitro* ESR detection using CMH (25 µg/ml) as spin label in isolated rat PBMC **(I)** and PMN **(J)** after incubation with p-/mCRP (10 µg/ml) ± prior treatment with nystatin (25 µg/ml) intending to disrupt lipid rafts. LPS (50 µg/ml) served as positive control. Values are mean ± SEM of eight different rats. ^#^*p* < 0.05 compared to the control group. *p < 0.05 for mCRP vs. mCRP + nystatin. Disruption of lipid rafts by nystatin prior to incubation with mCRP significantly decreases ROS generation in PBMC. Signal drop did not reach statistical significance in PMN.

Generation of ROS was assessed *ex vivo* in rat PBMC (Figure 4A) and PMN (Figure 4B) *via* ESR spectroscopy. In both leukocyte subsets, intravenous treatment with mCRP significantly increases ROS formation. In contrast, the infusion of pCRP showed no such effect. This is in line with the *in vitro* stimulation of previously isolated PBMC (Figure 4C) and PMN (Figure 4D) where mCRP also significantly induces ROS formation. These results can be reproduced for human isolated leukocytes (Figures 4E,F). Figure 4G shows representative ESR spectra. To investigate the significance of lipid rafts in the CRP-mediated respiratory burst, isolated PBMC and PMN were treated with Nystatin prior to incubation with CRP. ESR spectroscopy reveals that disruption of lipid rafts on PBMC masks the pro-inflammatory potential of mCRP resulting in a significant decrease in ROS formation (Figure 4I). On PMN, however, treatment with Nystatin failed to eliminate the pro-inflammatory potential of mCRP (Figure 4J) indicating alternative signaling pathways.

### CRP Is Deposited in the IRI of Human Striated Muscle Tissue and Co-Localizes With CD68^+^ Leukocytes

**Figure 5:**
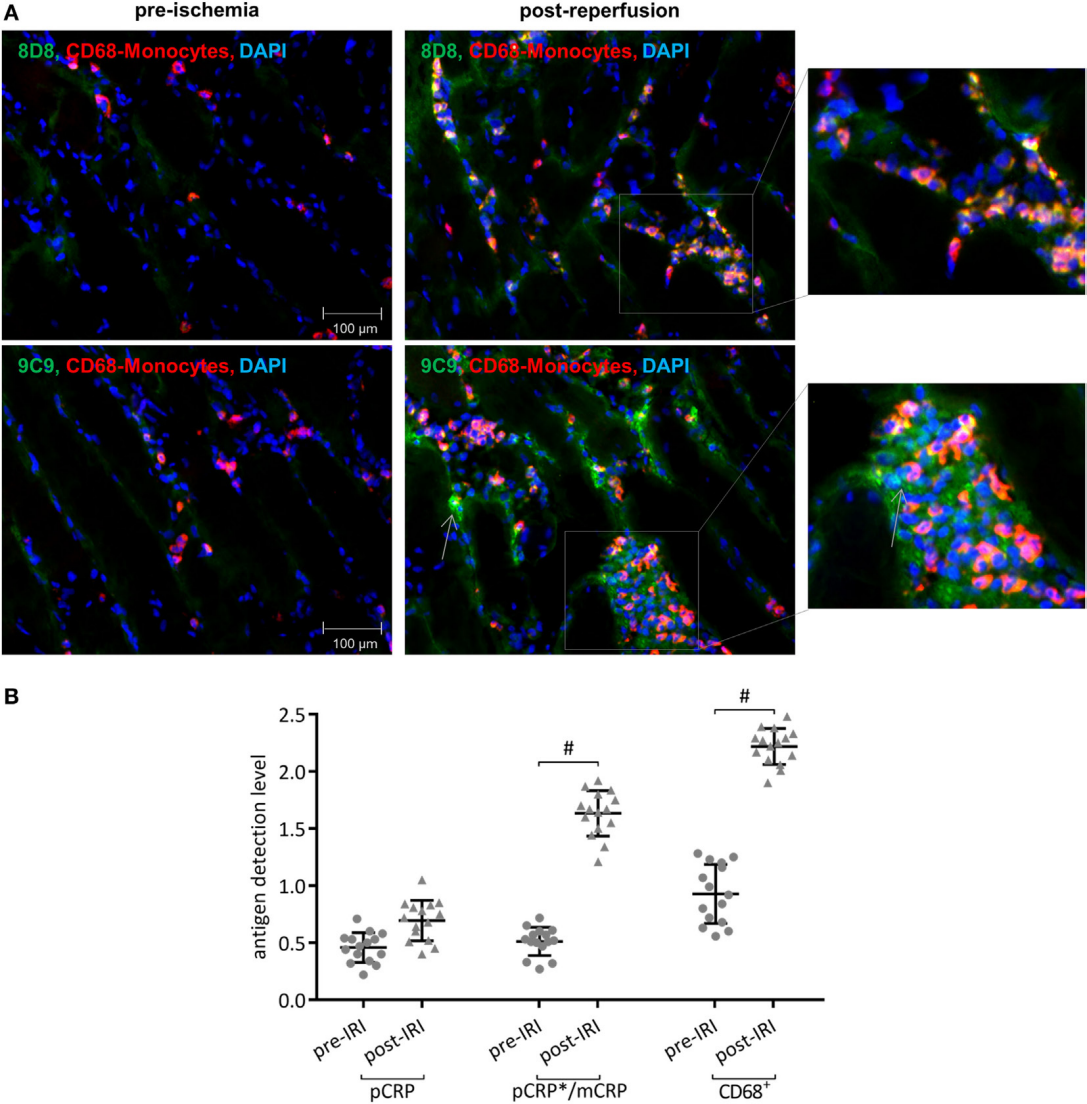
Conformationally altered C-reactive protein (CRP) accumulates in inflamed human muscle tissue. **(A)** Immunofluorescence conformation-specific detection of C-reactive protein and CD68^+^ cells in striated human muscle tissue shortly before (pre-ischemia) and after free tissue transfer (post-reperfusion). Conformation-specific antibody clone 8D8, which recognizes exclusively pentameric C-reactive protein (pCRP) and clone 9C9, recognizing neo-epitopes expressed by pCRP*/monomeric C-reactive protein (mCRP), were used. Typical results are given. **(B)** Quantification shows relative values of immunoreactivity for CD68^+^ cells and pCRP and pCRP*/mCRP, respectively. At least three non-overlapping images were evaluated from each sample to determine the corresponding value. ^#^*p* < 0.05 for pre-ischemia vs. ischemia/reperfusion injury (IRI). Values are mean ± SEM; *n* = 15. IRI leads to a significant increase of p- and especially conformationally altered CRP deposition, which co-localizes with CD68^+^ leukocytes.

Immunofluorescence with conformation specific detection of CRP and CD68^+^ cells in striated human muscle tissue shortly before (pre-ischemia) and after free tissue transfer (post-reperfusion) revealed extensive deposition of neo-epitope expressing CRP in the IRI-challenged tissue (Figure 5A). pCRP is detected in the reperfused muscle; however, deposition shows no significant increase when compared to pre-ischemic values. Elevated numbers of CD68^+^ leukocytes co-localize with neo-epitope expressing CRP in the inflamed tissue (Figure 5B).

## Discussion

Here, we identify and characterize the role of CRP in the pathological cascade of IRI by two distinct *in vivo* models of IRI, renal IRI, and IRI of striated muscle tissue. We further analyze the underlying mechanisms of CRP mediated tissue damage. Based on our findings, inflammatory tissue impairment in IRI can potentially be targeted by the prevention of molecular changes in CRP structure. This is supported by the following findings: (1) CRP aggravates renal IRI in a rat model and aggravates ischemia induced renal damage. (2) CRP undergoes a conformational change in renal IRI leading to exposure of pro-inflammatory epitopes (pCRP*/mCRP). (3) mCRP induces significant leukocyte activation in the microcirculation of the rat cremaster muscle. (4) Accumulated CRP in IRI consists mostly of conformationally altered isoforms. (5) The pro-inflammatory potential of CRP in renal IRI and IRI of striated muscle tissue can be blocked by preventing the conformational change of pCRP. (6) mCRP, but not pCRP, induces ROS generation *in vivo* and *ex vivo*. (7) mCRP-mediated ROS formation in PBMC is mediated by lipid rafts. (8) In human IRI of striated muscle tissue, neo-epitope expressing CRP accumulates and co-localizes with inflammatory cells, suggesting a transferability of our results into the *in vivo* situation in humans.

There is a growing body of evidence suggesting a causal role for CRP in IRI. Padilla and coworkers were able to show that CRP is an activator of complement in a rat model of intestinal IRI (38). Another report revealed that CRP exacerbates renal IRI in transgenic human CRP mice compared to a wild-type control (39). Only recently, the same group showed in their mouse model that myeloid-derived suppressor cells might participate in the CRP-driven inflammation in renal IRI (40). Our work provides the underlying mechanism by which CRP contributes to IRI. We are able to show that pCRP does not exert any pro-inflammatory effects, which is in line with previous findings (41–43). Moreover, we can show that a paramount requirement for the aggravation of inflammation by CRP is the expression of the pro-inflammatory neo-epitope, thus defining the molecular basis for the effects observed in the aforementioned publications. A rat model was used, as even though rats have abundant CRP (300–600 µg/ml in normal healthy pathogen-free rats), it does not activate rat complement. This is in contrast to human CRP that, similar to the *in vivo* situation in humans, activates rat complement and thus makes the rat the ideal animal model for CRP research (44).

We have previously shown that the conformational change of circulating pCRP is a localized process limited to the area of inflammation (11) and is mediated by activated cell membranes carrying bioactive lipids, such as lysophosphatidylcholine (10, 15). In our recent work, we identify an initial structural change in the pentameric protein (pCRP*) after binding to activated monocytes that leads to the expression of pro-inflammatory neo-epitopes resembling those of mCRP (12). The pathophysiological cascade from pCRP binding to activated membranes, consecutive generation of pCRP* and finally mCRP formation has proven its pro-inflammatory potential *in vitro* (12) and in an *in vivo* model of acute inflammation (11). Here, we demonstrate the significance of structural alterations in CRP for the first time in clinically highly relevant renal IRI. Inhibiting the conformational change of pCRP with a compound that stabilizes pCRP in a decameric conformation, first described by Pepys et al. (45) abrogates all CRP effects. We have recently shown that in this decameric conformation, CRP is not able to undergo its conformational change with exposure of pro-inflammatory epitopes (11, 46). In the IRI models that we investigated herein, 1,6-bisPC blunts the pro-inflammatory effects of CRP that promoted an aggravated inflammatory reaction beforehand. 1,6-bisPC itself, however, shows no intrinsic anti-inflammatory potential. We thereby confirm the conformational change as “*conditio sine qua non*” for the pro-inflammatory properties and provide prove of the feasibility of therapeutically locking pCRP in its native isoform in order to attenuate IRI-induced tissue damage.

The mCRP-induced ROS generation *in vitro* is a significant finding of our work, which is also confirmed *ex vivo*. The formation of oxygen radicals is of causal relevance in various diseases, such as atherosclerosis (47), myocardial infarction (48, 49), and other inflammatory diseases (50). We investigated the generation of ROS *ex vivo* in leukocytes by ESR spectroscopy following *in vivo* exposition to p-/mCRP, thereby indicating leukocyte activation and oxidative stress. This was further supported by the *in vitro* analysis of CRP-induced radical formation in leukocytes. Our findings demonstrate that mCRP induces oxidative stress in different leukocyte subsets, which potentially aggravates tissue damage in the course of IRI associated inflammation.

Recent literature proposes mCRP–lipid raft interaction as an important mechanism in mediating cellular responses to mCRP in human cells (Figure 4H) (10, 51). Lipid rafts represent dynamic, detergent-resistant plasma membrane microdomains that are highly enriched in cholesterol and sphingolipids and play critical roles in cellular signaling (52, 53). For the interaction of mCRP with lipid rafts, a direct membrane integration of mCRP has been proposed (51). At the same time, the FcγR-I can be found in lipid raft microdomains (54) and has previously been identified as a potent pro-inflammatory mediator of mCRP on human monocytes (11) and potent inductor for ROS formation (55) *via* induction of NADPH oxidases (56). Disruption of lipid rafts with nystatin abrogates the potential binding sites for mCRP in lipid rafts microdomains. This inhibits the mCRP-induced respiratory burst in PBMC, however, fails to abrogate the pro-inflammatory CRP effects in PMN. This might be explained through distinct receptor patches on different leukocyte subsets such as a higher significance of FcγR-IIIa in PMN that has shown to mediate mCRP signaling (11). The inhibition of mCRP signal transduction by nystatin in PBMC, which does not inhibit LPS-induced generation of ROS, furthermore confirms the specificity of the mCRP effects, as it rules out potential influences of contaminating bacterial products as described by other authors (57).

**Figure 6:**
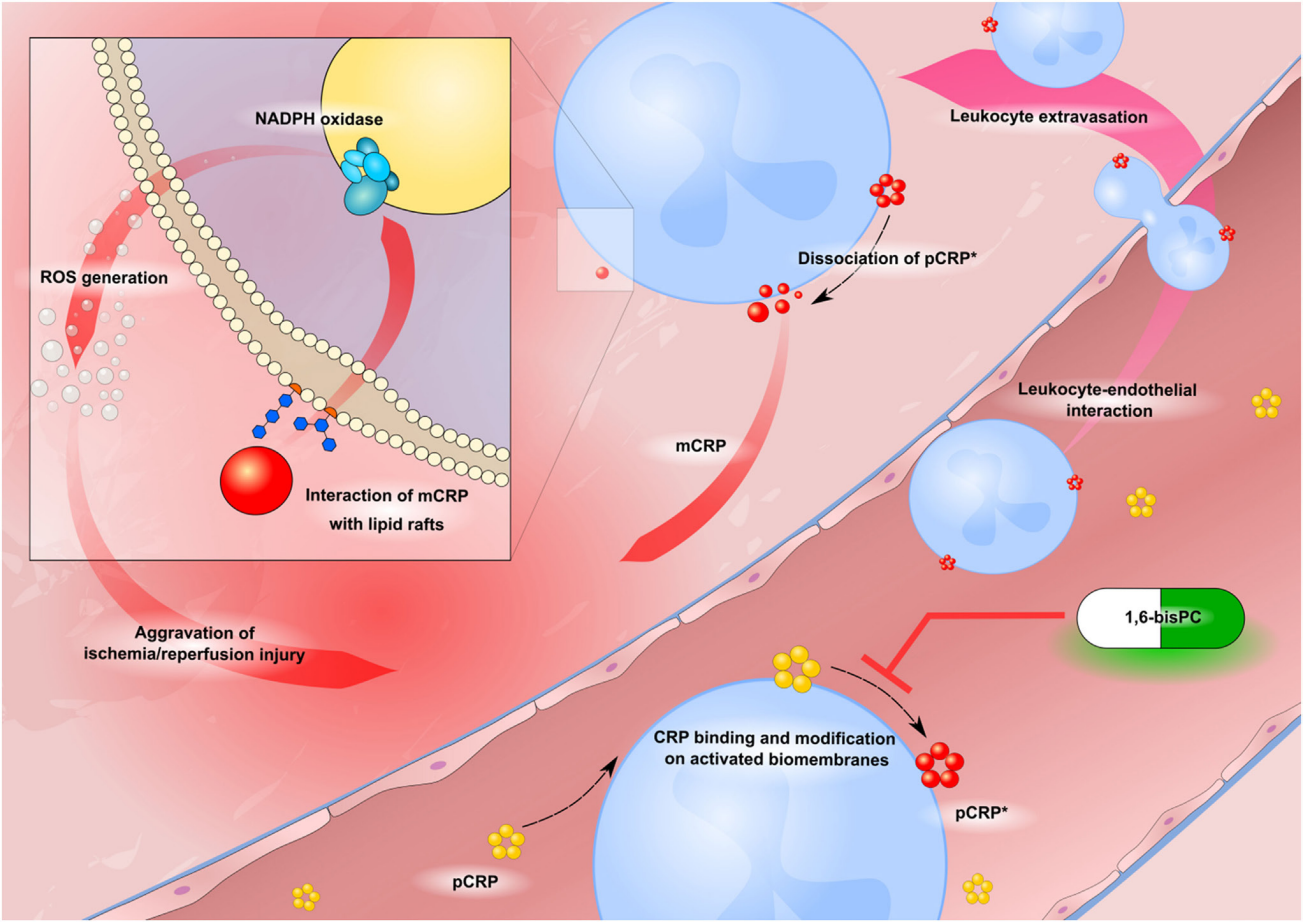
Schematic drawing of CRP-driven leukocyte response in ischemia/reperfusion injury (IRI). Circulating pentameric C-reactive protein (pCRP) (yellow) binds to activated biomembranes in the microcirculation of inflamed tissue. It is subsequently conformationally altered to bioactive pCRP*, dissociates and forms mCRP (red circles). Neo-epitope expressing CRP induces leukocyte–endothelial interaction and activation of the ROS producing NADPH oxidase enzyme complex. 1,6-bisPC (green–white pill) prevents the CRP-mediated leukocyte activation by stabilizing the native pentameric isoform of CRP.

In conclusion, we demonstrate that CRP aggravates IRI *via* various pro-inflammatory mechanisms. Formation of neo-epitope expressing CRP leads to significant renal damage and induces leukocyte–endothelial interaction and generation of ROS. These effects are in part mediated by lipid raft signaling and can be therapeutically targeted by blocking pCRP dissociation with 1,6-bisPC (Figure 6).

## Ethics Statement

This study was carried out in accordance with the recommendations of the animal ethic committee of the University of Freiburg Medical Center, Germany. The protocol was approved by the animal ethic committee of the University of Freiburg Medical Center, Germany. For immunohistology of human ischemia/reperfusion-injured tissue, biopsies of 15 patients receiving free muscle flap reconstruction of posttraumatic soft tissue defects of the lower extremity were taken between September 2008 and March 2010. Informed consent was obtained from each patient. The study was approved by the ethic committee of the University of Freiburg Medical Centre (Application number: 67/08) and conducted in accordance with the declaration of Helsinki.

## Author Contributions

JT: conducted main part of experiments and authored the manuscript with JZ. JZ: conducted main part of experiments and authored the manuscript with JT. JK, DB, and YL, HB, and KP: contributed to the experiments and the authoring of the manuscript. SK: provided support in lab work and contributed to the experiments. LP: provided conformation-specific CRP antibodies. FG: supported in establishment of renal IRI model and contributed to the authoring of the manuscript. TH: supported in establishment of renal IRI model and contributed to the authoring of the manuscript. MH-L: contributed to the interpretation of data, authoring and final approval of the manuscript. GS: contributed to the authoring of the manuscript. SE: planned the experimental procedures, contributed in main parts to the experiments, and the authoring of the manuscript.

## Conflict of Interest Statement

The authors declare that the research was conducted in the absence of any commercial or financial relationships that could be construed as a potential conflict of interest.

## Abbreviations

CRP, C-reactive protein; mCRP, monomeric C-reactive protein; pCRP, pentameric C-reactive protein; pCRP*, neo-epitope expressing CRP; IRI, ischemia/reperfusion injury; 1,6-bisPC, 1,6-bis(phosphocholine)-hexane; PBMC, peripheral blood mononuclear cells; PMN, polymorphonuclear cells; D-PBS, Dulbecco’s physiological phosphate-buffered saline; NADPH, reduced nicotinamide adenine dinucleotide phosphate.

## References

[1] Cerra FB Lajos TZ Montes M Siegel JH. Hemorrhagic infarction: a reperfusion injury following prolonged myocardial ischemic anoxia. Surgery (1975) 78(1):95–104. 1138403

[2] Farhood A McGuire GM Manning AM Miyasaka M Smith CW Jaeschke H. Intercellular adhesion molecule 1 (ICAM-1) expression and its role in neutrophil-induced ischemia-reperfusion injury in rat liver. J Leukoc Biol (1995) 57(3):368–74. 10.1002/jlb.57.3.368 7884306

[3] Lehr HA Guhlmann A Nolte D Keppler D Messmer K. Leukotrienes as mediators in ischemia-reperfusion injury in a microcirculation model in the hamster. J Clin Invest (1991) 87(6):2036–41. 10.1172/JCI115233 1645749 PMC296959

[4] Lazarus B Messina A Barker JE Hurley JV Romeo R Morrison WA The role of mast cells in ischaemia-reperfusion injury in murine skeletal muscle. J Pathol (2000) 191(4):443–8. 10.1002/1096-9896(2000)9999:9999<::AID-PATH666>3.0.CO;2-L 10918220

[5] Rashid MA William-Olsson G. Are leukocytosis and lipid peroxidation involved in ischemic or reperfusion injury in cardiac surgery? Thorac Cardiovasc Surg (1991) 39(4):193–5. 10.1055/s-2007-1022707 1948967

[6] Zimmerman BJ Granger DN Oxygen free radicals and the gastrointestinal tract: role in ischemia-reperfusion injury. Hepatogastroenterology (1994) 41(4):337–42. 7959568

[7] Wang WZ Fang XH Stephenson LL Khiabani KT Zamboni WA. Melatonin reduces ischemia/reperfusion-induced superoxide generation in arterial wall and cell death in skeletal muscle. J Pineal Res (2006) 41(3):255–60. 10.1111/j.1600-079X.2006.00361.x 16948786

[8] Gobé G Willgoss D Hogg N Schoch E Endre Z. Cell survival or death in renal tubular epithelium after ischemia-reperfusion injury. Kidney Int (1999) 56(4):1299–304. 10.1046/j.1523-1755.1999.00701.x 10504480

[9] Perico N Cattaneo D Sayegh MH Remuzzi G. Delayed graft function in kidney transplantation. Lancet (2004) 364(9447):1814–27. 10.1016/S0140-6736(04)17406-0 15541456

[10] Eisenhardt SU Habersberger J Murphy A Chen YC Woollard KJ Bassler N Dissociation of pentameric to monomeric C-reactive protein on activated platelets localizes inflammation to atherosclerotic plaques. Circ Res (2009) 105(2):128–37. 10.1161/CIRCRESAHA.108.190611 19520972

[11] Thiele JR Habersberger J Braig D Schmidt Y Goerendt K Maurer V Dissociation of pentameric to monomeric C-reactive protein localizes and aggravates inflammation: in vivo proof of a powerful proinflammatory mechanism and a new anti-inflammatory strategy. Circulation (2014) 130(1):35–50. 10.1161/CIRCULATIONAHA.113.007124 24982116

[12] Braig D Nero TL Koch HG Kaiser B Wang X Thiele JR Transitional changes in the CRP structure lead to the exposure of proinflammatory binding sites. Nat Commun (2017) 8:14188. 10.1038/ncomms14188 28112148 PMC5264208

[13] Braig D Kaiser B Thiele JR Bannasch H Peter K Stark GB A conformational change of C-reactive protein in burn wounds unmasks its proinflammatory properties. Int Immunol (2014) 26(8):467–78. 10.1093/intimm/dxu056 24844702

[14] Molins B Fuentes-Prior P Adán A Antón R Arostegui JI Yagüe J Complement factor H binding of monomeric C-reactive protein downregulates proinflammatory activity and is impaired with at risk polymorphic CFH variants. Sci Rep (2016) 6:22889. 10.1038/srep22889 26961257 PMC4785391

[15] Ji SR Wu Y Zhu L Potempa LA Sheng FL Lu W Cell membranes and liposomes dissociate C-reactive protein (CRP) to form a new, biologically active structural intermediate: mCRP(m). FASEB J (2007) 21(1):284–94. 10.1096/fj.06-6722com 17116742

[16] Eisenhardt SU Thiele JR Bannasch H Stark GB Peter K. C-reactive protein: how conformational changes influence inflammatory properties. Cell Cycle (2009) 8(23):3885–92. 10.4161/cc.8.23.10068 19887916

[17] Slevin M Matou-Nasri S Turu M Luque A Rovira N Badimon L Modified C-reactive protein is expressed by stroke neovessels and is a potent activator of angiogenesis in vitro. Brain Pathol (2010) 20(1):151–65. 10.1111/j.1750-3639.2008.00256.x 19170684 PMC8094831

[18] de la Torre R Peña E Vilahur G Slevin M Badimon L. Monomerization of C-reactive protein requires glycoprotein IIb-IIIa activation: pentraxins and platelet deposition. J Thromb Haemost (2013) 11(11):2048–58. 10.1111/jth.12415 24119011

[19] Khreiss T József L Hossain S Chan JS Potempa LA Filep JG. Loss of pentameric symmetry of C-reactive protein is associated with delayed apoptosis of human neutrophils. J Biol Chem (2002) 277(43):40775–81. 10.1074/jbc.M205378200 12198121

[20] Potempa LA Yao ZY Ji SR Filep JG Wu Y. Solubilization and purification of recombinant modified C-reactive protein from inclusion bodies using reversible anhydride modification. Biophys Rep (2015) 1:18–33. 10.1007/s41048-015-0003-2 26942216 PMC4762138

[21] Delbridge MS Shrestha BM Raftery AT El Nahas AM Haylor JL. The effect of body temperature in a rat model of renal ischemia-reperfusion injury. Transplant Proc (2007) 39(10):2983–5. 10.1016/j.transproceed.2007.04.028 18089305

[22] Wei Q Dong Z Mouse model of ischemic acute kidney injury: technical notes and tricks. Am J Physiol Renal Physiol (2012) 303(11):F1487–94. 10.1152/ajprenal.00352.2012 22993069 PMC3532486

[23] Curtin LI Grakowsky JA Suarez M Thompson AC DiPirro JM Martin LB Evaluation of buprenorphine in a postoperative pain model in rats. Comp Med (2009) 59(1):60–71. 19295055 PMC2703135

[24] Guarnieri M Brayton C DeTolla L Forbes-McBean N Sarabia-Estrada R Zadnik P. Safety and efficacy of buprenorphine for analgesia in laboratory mice and rats. Lab Anim (NY) (2012) 41(11):337–43. 10.1038/laban.152 23079917

[25] Lee HB Blaufox MD. Blood volume in the rat. J Nucl Med (1985) 26(1):72–6. 3965655

[26] Nishiki T Kitada H Okabe Y Miura Y Kurihara K Kawanami S Effect of milrinone on ischemia-reperfusion injury in the rat kidney. Transplant Proc (2011) 43(5):1489–94. 10.1016/j.transproceed.2011.03.009 21693223

[27] Williams P Lopez H Britt D Chan C Ezrin A Hottendorf R. Characterization of renal ischemia-reperfusion injury in rats. J Pharmacol Toxicol Methods (1997) 37(1):1–7. 10.1016/S1056-8719(96)00141-4 9086282

[28] Strang F Scheichl A Chen YC Wang X Htun NM Bassler N Amyloid plaques dissociate pentameric to monomeric C-reactive protein: a novel pathomechanism driving cortical inflammation in Alzheimer’s disease? Brain Pathol (2012) 22(3):337–46. 10.1111/j.1750-3639.2011.00539.x 21951392 PMC8092962

[29] Fuzio P Ditonno P Rutigliano M Battaglia M Bettocchi C Loverre A Regulation of TGF-β1 expression by androgen deprivation therapy of prostate cancer. Cancer Lett (2012) 318(2):135–44. 10.1016/j.canlet.2011.08.034 22269108

[30] Barbe MF Barr AE Gorzelany I Amin M Gaughan JP Safadi FF. Chronic repetitive reaching and grasping results in decreased motor performance and widespread tissue responses in a rat model of MSD. J Orthop Res (2003) 21(1):167–76. 10.1016/S0736-0266(02)00086-4 12507595 PMC1560095

[31] Megyesi J Andrade L Vieira JM Jr Safirstein RL Price PM. Positive effect of the induction of p21WAF1/CIP1 on the course of ischemic acute renal failure. Kidney Int (2001) 60(6):2164–72. 10.1046/j.1523-1755.2001.00044.x 11737590

[32] Megyesi J Safirstein RL Price PM. Induction of p21WAF1/CIP1/SDI1 in kidney tubule cells affects the course of cisplatin-induced acute renal failure. J Clin Invest (1998) 101(4):777–82. 10.1172/JCI1497 9466972 PMC508625

[33] Graus-Nunes F Marinho TS Barbosa-da-Silva S Aguila MB Mandarim-de-Lacerda CA Souza-Mello V. Differential effects of angiotensin receptor blockers on pancreatic islet remodelling and glucose homeostasis in diet-induced obese mice. Mol Cell Endocrinol (2017) 439:54–64. 10.1016/j.mce.2016.10.021 27780713

[34] Ying SC Gewurz H Kinoshita CM Potempa LA Siegel JN. Identification and partial characterization of multiple native and neoantigenic epitopes of human C-reactive protein by using monoclonal antibodies. J Immunol (1989) 143(1):221–8. 2471736

[35] Thiele JR Goerendt K Stark GB Eisenhardt SU. Real-time digital imaging of leukocyte-endothelial interaction in ischemia-reperfusion injury (IRI) of the rat cremaster muscle. J Vis Exp (2012) (66):e3973. 10.3791/3973 22895495 PMC3476763

[36] Baatz H Steinbauer M Harris AG Krombach F. Kinetics of white blood cell staining by intravascular administration of rhodamine 6G. Int J Microcirc Clin Exp (1995) 15(2):85–91. 10.1159/000178955 8655257

[37] Kuzkaya N Weissmann N Harrison DG Dikalov S. Interactions of peroxynitrite with uric acid in the presence of ascorbate and thiols: implications for uncoupling endothelial nitric oxide synthase. Biochem Pharmacol (2005) 70(3):343–54. 10.1016/j.bcp.2005.05.009 15963955

[38] Padilla ND van Vliet AK Schoots IG Valls Seron M Maas MA Peltenburg EE C-reactive protein and natural IgM antibodies are activators of complement in a rat model of intestinal ischemia and reperfusion. Surgery (2007) 142(5):722–33. 10.1016/j.surg.2007.05.015 17981193

[39] Pegues MA McCrory MA Zarjou A Szalai AJ C-reactive protein exacerbates renal ischemia-reperfusion injury. Am J Physiol Renal Physiol (2013) 304(11):F1358–65. 10.1152/ajprenal.00476.2012 23535585 PMC3680688

[40] Pegues MA McWilliams IL Szalai AJ. C-reactive protein exacerbates renal ischemia-reperfusion injury: are myeloid-derived suppressor cells to blame? Am J Physiol Renal Physiol (2016) 311(1):F176–81. 10.1152/ajprenal.00107.2016 27053688 PMC4967164

[41] Eisenhardt SU Starke J Thiele JR Murphy A Bjorn Stark G Bassler N Pentameric CRP attenuates inflammatory effects of mmLDL by inhibiting mmLDL—monocyte interactions. Atherosclerosis (2012) 224(2):384–93. 10.1016/j.atherosclerosis.2012.07.039 22901456

[42] Lane T Wassef N Poole S Mistry Y Lachmann HJ Gillmore JD Infusion of pharmaceutical-grade natural human C-reactive protein is not proinflammatory in healthy adult human volunteers. Circ Res (2014) 114(4):672–6. 10.1161/CIRCRESAHA.114.302770 24337102

[43] Pepys MB Gallimore JR Lloyd J Li Z Graham D Taylor GW Isolation and characterization of pharmaceutical grade human pentraxins, serum amyloid P component and C-reactive protein, for clinical use. J Immunol Methods (2012) 384(1–2):92–102. 10.1016/j.jim.2012.07.013 22867744 PMC4068106

[44] de Beer FC Baltz ML Munn EA Feinstein A Taylor J Bruton C Isolation and characterization of C-reactive protein and serum amyloid P component in the rat. Immunology (1982) 45(1):55–70. 7056568 PMC1555156

[45] Pepys MB Hirschfield GM Tennent GA Gallimore JR Kahan MC Bellotti V Targeting C-reactive protein for the treatment of cardiovascular disease. Nature (2006) 440(7088):1217–21. 10.1038/nature04672 16642000

[46] Habersberger J Strang F Scheichl A Htun N Bassler N Merivirta RM Circulating microparticles generate and transport monomeric C-reactive protein in patients with myocardial infarction. Cardiovasc Res (2012) 96(1):64–72. 10.1093/cvr/cvs237 22798388

[47] Woollard KJ Geissmann F. Monocytes in atherosclerosis: subsets and functions. Nat Rev Cardiol (2010) 7(2):77–86. 10.1038/nrcardio.2009.228 20065951 PMC2813241

[48] Luo X Cai H Ni J Bhindi R Lowe HC Chesterman CN c-Jun DNAzymes inhibit myocardial inflammation, ROS generation, infarct size, and improve cardiac function after ischemia-reperfusion injury. Arterioscler Thromb Vasc Biol (2009) 29(11):1836–42. 10.1161/ATVBAHA.109.189753 19592465

[49] Vasilyev N Williams T Brennan ML Unzek S Zhou X Heinecke JW Myeloperoxidase-generated oxidants modulate left ventricular remodeling but not infarct size after myocardial infarction. Circulation (2005) 112(18):2812–20. 10.1161/CIRCULATIONAHA.105.542340 16267254

[50] Gill R Tsung A Billiar T. Linking oxidative stress to inflammation: toll-like receptors. Free Radic Biol Med (2010) 48(9):1121–32. 10.1016/j.freeradbiomed.2010.01.006 20083193 PMC3423196

[51] Ji SR Ma L Bai CJ Shi JM Li HY Potempa LA Monomeric C-reactive protein activates endothelial cells via interaction with lipid raft microdomains. FASEB J (2009) 23(6):1806–16. 10.1096/fj.08-116962 19136614

[52] Lingwood D Simons K Lipid rafts as a membrane-organizing principle. Science (2010) 327(5961):46–50. 10.1126/science.1174621 20044567

[53] Simons K Toomre D Lipid rafts and signal transduction. Nat Rev Mol Cell Biol (2000) 1(1):31–9. 10.1038/35036052 11413487

[54] Beekman JM van der Linden JA van de Winkel JG Leusen JH. FcgammaRI (CD64) resides constitutively in lipid rafts. Immunol Lett (2008) 116(2):149–55. 10.1016/j.imlet.2007.12.003 18207250

[55] Newbrough SA Mocsai A Clemens RA Wu JN Silverman MA Singer AL SLP-76 regulates Fcgamma receptor and integrin signaling in neutrophils. Immunity (2003) 19(5):761–9. 10.1016/S1074-7613(03)00305-4 14614862

[56] Pfefferkorn LC Fanger MW. Transient activation of the NADPH oxidase through Fc gamma RI. Oxidase deactivation precedes internalization of cross-linked receptors. J Immunol (1989) 143(8):2640–9. 2551966

[57] Pepys MB Hawkins PN Kahan MC Tennent GA Gallimore JR Graham D Proinflammatory effects of bacterial recombinant human C-reactive protein are caused by contamination with bacterial products, not by C-reactive protein itself. Circ Res (2005) 97(11):e97–103. 10.1161/01.RES.0000193595.03608.08 16254214 PMC1400607

